# Biomechanical evaluation of three implants for treating unstable femoral intertrochanteric fractures: finite element analysis in axial, bending and torsion loads

**DOI:** 10.3389/fbioe.2023.1279067

**Published:** 2023-11-07

**Authors:** HuanAn Bai, Lu Liu, Ning Duan, HanZhong Xue, Liang Sun, Ming Li, Zhong Li, Kun Zhang, Qian Wang, Qiang Huang

**Affiliations:** Department of Orthopedics, Hong Hui Hospital, Xi’an Jiaotong University, Xi’an, Shaanxi, China

**Keywords:** biomechanical, unstable, intertrochanteric fracture, finite element model, implant, femur

## Abstract

**Purpose:** How to effectively enhance the mechanical stability of intramedullary implants for unstable femoral intertrochanteric fractures (UFIFs) is challenging. The authors developed a new implant for managing such patients. Our aim was to enhance the whole mechanical stability of internal devices through increasing antirotation and medial support. We expected to reduce stress concentration in implants. Each implant was compared to proximal femoral nail antirotation (PFNA) via finite element method.

**Methods:** Adult AO/OTA 31-A2.3 fracture models were constructed, and then the new intramedullary system (NIS), PFNA, InterTan nail models were assembled. We simulated three different kinds of load cases, including axial, bending, and torsion loads. For further comparison of PFNA and the NIS, finite element analysis (FEA) was repeated for five times under axial loads of 2100 N. Two types of displacement and stress distribution were assessed.

**Results:** Findings showed that the NIS had the best mechanical stability under axial, bending, and torsion load conditions compared to PFNA and InterTan. It could be seen that the NIS displayed the best properties with respect to maximal displacement while PFNA showed the worst properties for the same parameter in axial loads of 2100 N. In terms of maximal stress, also the NIS exhibited the best properties while PFNA showed the worst properties in axial loads of 2100 N. For bending and torsion load cases, it displayed a similar trend with that of axial loads. Moreover, under axial loads of 2100 N, the difference between the PFNA group and the NIS group was statistically significant (*p* < 0.05).

**Conclusion:** The new intramedullary system exhibited more uniform stress distribution and better biomechanical properties compared to the PFNA and InterTan. This might provide a new and efficacious device for managing unstable femoral intertrochanteric fractures.

## Introduction

Accompanied by the progress of population aging, the morbidity for hip fractures is rising every year. It is predicted to increase about 12% from 2010 to 2030 (Hung et al., 2012), and half of these patients will occur in Asia by 2050 (Long et al., 2022). 41%–50% of these fractures belong to femoral intertrochanteric fractures in elderly cases (Tanner et al., 2010). The mortality rate of such fractures could reach 36% within 1 year (Bhandari and Swiontkowski, 2017). The current recommended treatment plan is to undergo firm internal fixation within 24–48 h after injury (Kokoroghiannis et al., 2012). This is beneficial for patients to get early rehabilitation and avoid long-term bed rest complications, and current research recommends intramedullary fixation for most patients (Queally et al., 2014; Brox et al., 2015). However, the comminuted intertrochanteric fractures (especially AO/OTA 31-A2.3) account for more than 80% of UFIFs (Grønhaug et al., 2022), which are the main component of implant failure. The mechanical properties of a fixation device are the most important factors to guarantee good therapeutic effects and reduce implant failure.

**FIGURE 1:**
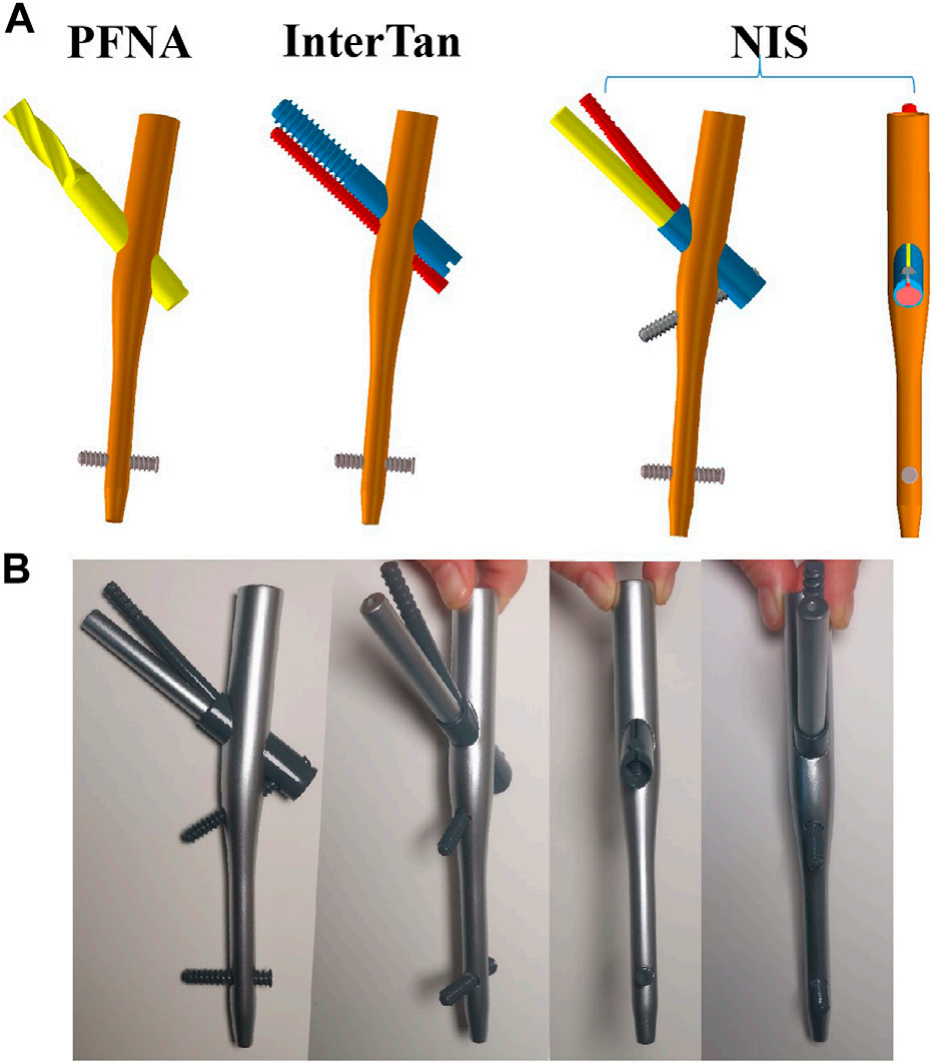
Three configurations when finite element models were assembled. **(A)** Schematic diagrams of three implants, including PFNA, InterTan, and NIS. **(B)** Metal model of the NIS. PFNA stands for proximal femoral nail antirotation. NIS stands for the new intramedullary system.

Currently, the widely used implants in treating UFIFs include PFNA, Gamma3 nails and InterTan nails. The fixation techniques of these intramedullary nails are minimally invasive and easy to operate. Yet, the proximal parts of UFIFs are prone to implant lossening and even failure because of swing effects while they are fixed via a cephalomedullary nail due to the osteoporotic medullary cavity resulting from osteopenia and the advanced age (Ceynowa et al., 2020; Chen et al., 2022; Song et al., 2022). The cephalomedullary nail of PFNA and Gamma3 is designed with only one nail, and its antirotation effect is poor. The InterTan nail has two parallel cephalomedullary nails at the neck, which can enhance its antirotation ability. However, the improvement of antirotation is limited. In addition, PFNA, Gamma3 and InterTan nails cannot provide enough medial support for patients with comminuted or defective medial wall of the femoral trochanter. The failure rate of these implants ranges from 8% to 56%, including withdrawal, cut-out, varus collapse, etc (Jin et al., 2005; Liu et al., 2010; Glassner and Tejwani, 2011; Chapman et al., 2018). Therefore, increasing antirotation and medial support of UFIFs are vital factors for lowering the incidence of implant failure (Li et al., 2019). For these reasons, our team developed a new intramedullary implant to manage UFIFs. The proximal section of the new implant contains three nails, with two cephalomedullary nails passing into the femoral head at a specific angle and one subtrochanteric nail supporting the medial wall (Figure 1). We supposed that this design might provide good antirotation and medial support for patients with UFIFs.

Finite element analysis is a computer simulation technique to simulate the real object via applying mathematic approximate values. It has gained widespread recognition in new implant designing field of traumatic orthopedics, such as allowing for precise quantitative calculation of displacement, and for load distributions in simulated new implants and relevant bones (Mahaisavariya et al., 2014; Jitprapaikulsarn al., 2021). For this study, a new intramedullary implant was designed to manage unstable femoral intertrochanteric fractures. The new intramedullary system (NIS) was compared to InterTan nail and PFNA via finite element method. The stress distribution and corresponding displacement were tested and recorded. Axial, bending, and torsion load conditions were simulated in the experiment. For further comparison of PFNA and the NIS, finite element analysis was repeated for five times under axial loads of 2100 N.

## Materials and methods

### Model construction and virtual surgery

This study was approved by the biomedical research ethics committee of the Xi’an Hong Hui hospital (No.202301006). Our team recruited a healthy male volunteer with the age of 65 years. The volunteer has presented written informed consent before participating in the research. A three-dimensional (3D) femoral model was built according to computed tomography (CT) scan data of the volunteer via Mimics software (Materialise, Leuven, Belgium). Voltage and current operating ranges were set to 70–140 kV and 30–800 mA for the CT equipment, respectively. Hounsfield Unit (HU) value was used to identify cortical bones. For cortical and cancellous bones, HU value was bounded by 700 (Abdul Wahab et al., 2020). Then, a standardized posteromedial unsupported UFIF model (AO/OTA 31-A2.3: the most unstable and common type among comminuted intertrochanteric fractures) was established according to previous studies (Li et al., 2019; Nie et al., 2022). The intertrochanteric crest and the lesser trochanter between the two osteotomy lines were removed, and part of the greater trochanter, especially the posterior part, was removed. Computer-aided design software was applied to construct three structures of fixation for UFIFs. After format conversion, the implant models were installed on the femurs. Three configurations were obtained: PFNA, InterTan nail and the NIS models. Figure 1A displays the schematic diagrams of three implants. Figure 1B shows the metal model of the new intramedullary system. For the NIS, the proximal part of the main nail is 17 mm (diameter). The specification of the main nail is 10 mm (diameter) × 170 mm (length). The specification of the sleeve, two cephalomedullary nails, and the subtrochanteric nail is 12 mm, 9 mm, 6.4 mm, and 5.0 mm in diameter for the NIS, respectively. In the NIS, the designed angle between the lower cephalomedullary nail and the main intramedullary nail is 130°. For the two cephalomedullary nails, it is 7.5°. Moreover, the designed angle between the subtrochanteric nail and the sleeve is 70°.

### Finite element settings

**FIGURE 2:**
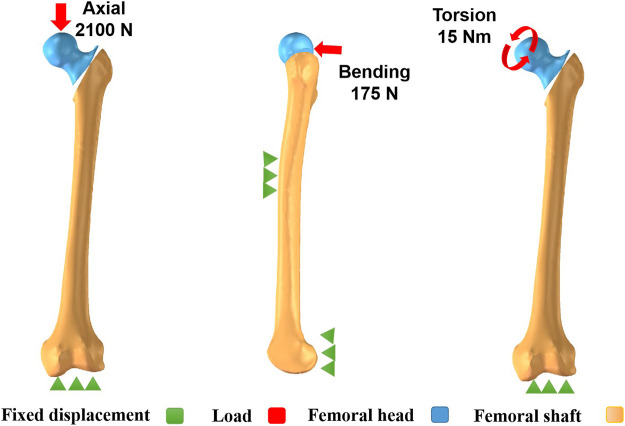
Boundary conditions for axial, bending, and torsion loads.

These three different configurations all were given homogeneous and linearly isotropic material characteristics. Tetrahedral elements were applied for meshing. To evaluate the reliability of three configurations, a convergence research was performed (Huang et al., 2023). In the case of maximum Degree of Freedom, the field parameters of the two types of elements, including strain energy and displacement, were in the range of 5%. In addition, the maximal stress point did not exist. Table 1 displayed the values of elements and nodes for three configuration models. In terms of material features, the elastic modulus was set at 16,800 MPa, 840 MPa, and 110,000 MPa for cortical, cancellous bones, and implants, respectively (Li et al., 2018; Huang et al., 2023). In terms of Poisson’s ratio, it was set at 0.3 with regard to cortical bones and implants while 0.2 as to cancellous bones (Li et al., 2018). These three implants were set to titanium material features as titanium and its alloys possess good biocompatibility, excellent corrosion resistance, and superior mechanical characteristics (Meng et al., 2022). Frictional contacts were defined for all contact conditions. The friction coefficient was set at 0.4 based on former studies (Viceconti et al., 2000). Figure 2 showed the boundary conditions for axial, bending, and torsion loads. The femoral condyle was tightly fixed to prohibit the overall motion of configurations. The axial loads were 2,100 N, acting vertically onto the surface of the femoral head (Zhang et al., 2011; Li et al., 2019). Under bending boundary conditions, the femoral shaft and condyle were properly fixed. The loads were set as 175 N, acting laterally onto the femoral head (Li et al., 2018). Under torsion boundary conditions, with the direction of the femoral neck as the axis, the torsion loads were set at 15 Nm acting onto the femoral head (Li et al., 2018).

**TABLE 1:** Number of nodes and elements for the three different implants.

Model	Nodes	Elements
PFNA	405406	261379
InterTan	506609	323206
NIS	494536	314645

PFNA, proximal femoral nail antirotation; NIS, new intramedullary system.

### Indexes for evaluation

Displacement and stress for implants and bones were recorded and assessed. PFNA was set as the control group as it has been widely used in clinical work recently and was recognized to possess superior mechanical properties (Lewis et al., 2022). The variation rate was estimated using this arithmetic formula: VR =(V_1_ − Vn)/V_1_×100%. VR stands for the variation rate. Vn stands for values of InterTan, or NIS. V_1_ means the value for PFNA. Besides, in order to compare PFNA and the NIS deeply, finite element analysis was repeated for five times under axial loads of 2100 N.

### Statistical analysis

This research used SPSS 23.0 software (IBM Co., United States) to perform statistical analysis. Finite element analysis data, including two types of displacement and stress under axial loads of 2100 N, between the PFNA and NIS groups were statistically compared using the Student’s t-test. *p* < 0.05 was defined as statistically significant.

## Results

### Maximal displacement for three fixation models

**FIGURE 3:**
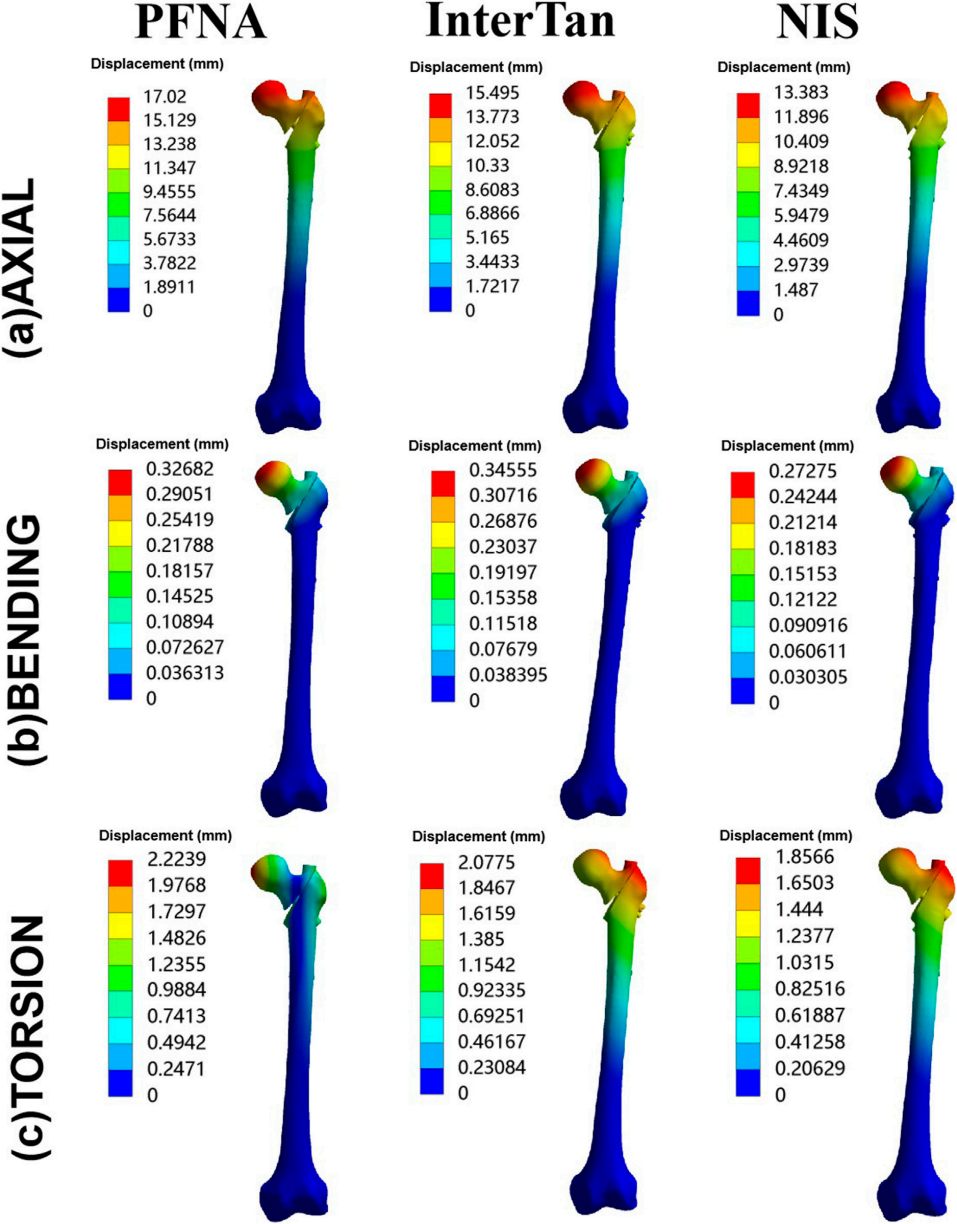
Maximal displacement of three configurations for axial, bending, and torsion loads. Three configurations included PFNA, InterTan, and NIS. PFNA stands for proximal femoral nail antirotation. NIS stands for the new intramedullary system.

Figure 3 showed maximal displacement of three configurations under different load conditions. Specially, when axial loads of 2100 N were applied the maximal displacement for PFNA, InterTan and NIS was 17.02 mm, 15.50 mm, and 13.38 mm (0.33 mm, 0.35 mm, and 0.27 mm under bending loads of 175 N; 2.22 mm, 2.08 mm, and 1.86 mm under torsion loads of 15 Nm). Compared with PFNA, the maximal displacement reduction for NIS was 21.4% under axial loads (16.5% under bending loads and 16.5% under torsion loads). The maximal displacement of the NIS was significantly lower compared to the value of PFNA.

### Maximal displacement of fracture surface (MDFS)

**FIGURE 4:**
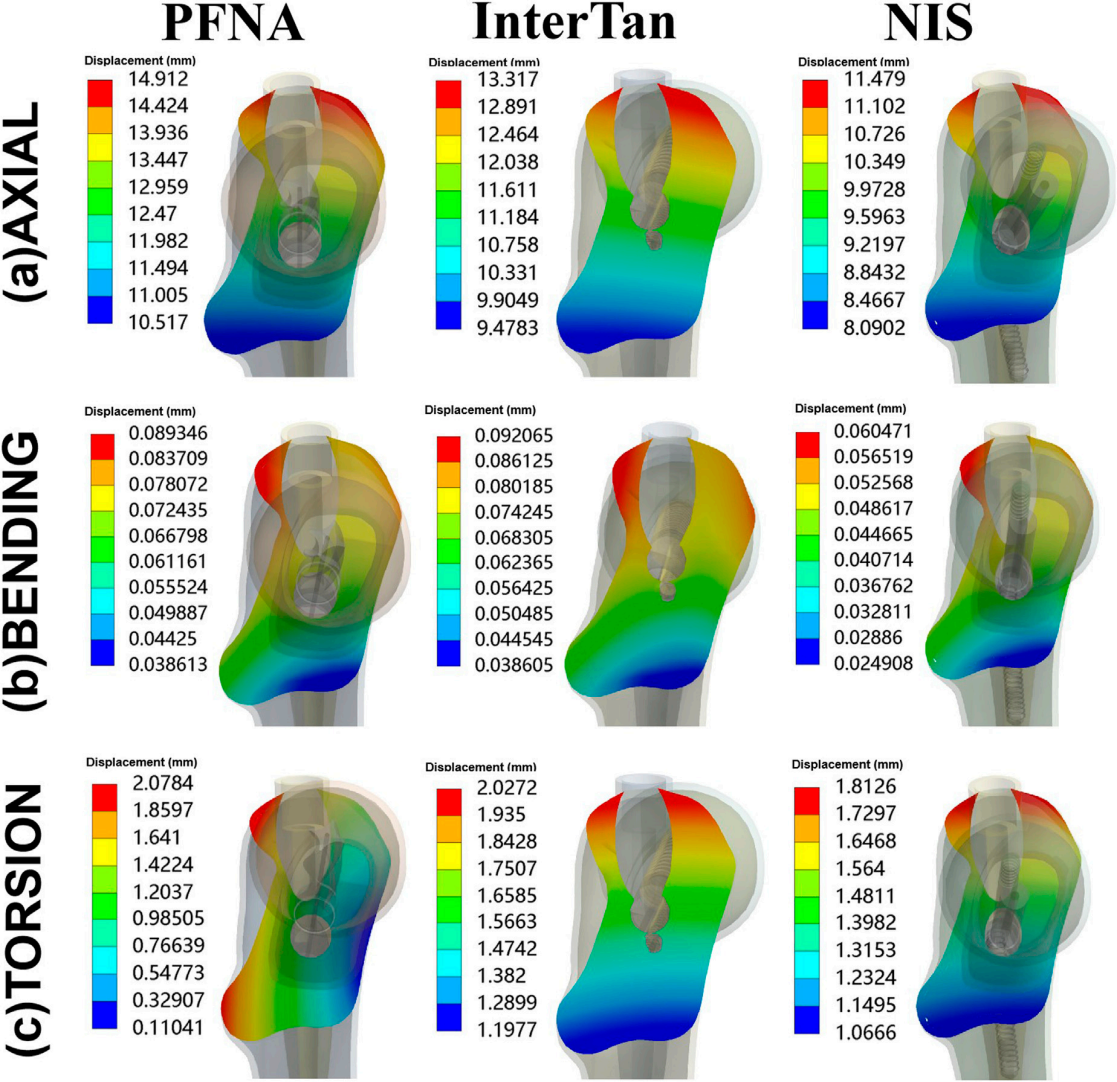
Maximal displacement of fracture surface for three configurations. Three configurations included PFNA, InterTan, and NIS. PFNA stands for proximal femoral nail antirotation. NIS stands for the new intramedullary system.

The values of MDFS for three different configurations were shown in Figure 4. While axial loads of 2100 N were applied, from the highest to the lowest value of this index, the three configurations were sorted as follows: PFNA, InterTan, and NIS. For bending load conditions, they were sorted as follows: InterTan, PFNA, and NIS. For torsion load conditions, they were sorted as follows: PFNA, InterTan, and NIS. The value of MDFS for the NIS was less than that of PFNA under three load cases. The MDFS reduction of the NIS relative to PFNA was 23.0% under axial loads of 2100 N while 32.3% for bending conditions. In addition, compared to PFNA, the MDFS reduction of the NIS was 12.8% when torsion loads were applied. The trend direction of MDFS was similar to that of maximal displacement for three fixation configurations. The above data indicated that the new intramedullary system possessed better biomechanical stability than PFNA for managing UFIFs.

### Stress at implants for three fixation models

**FIGURE 5:**
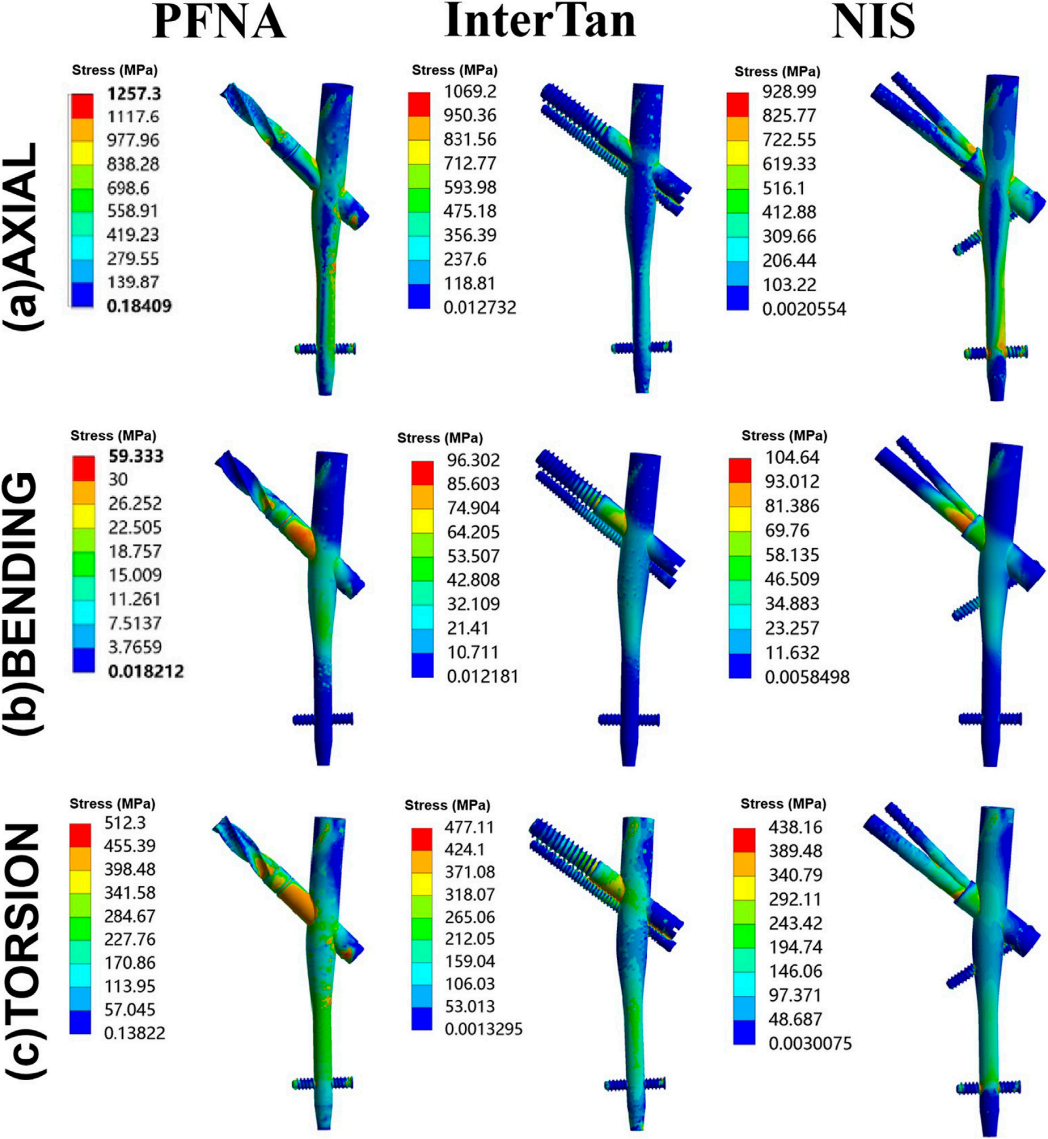
Von Mises stress on implants under axial, bending, and torsion loads for three configurations. Three configurations included PFNA, InterTan, and NIS. PFNA stands for proximal femoral nail antirotation. NIS stands for the new intramedullary system.

Figure 5 showed stress on implants for three fixation models. The maximal stress of the three implants occurred near the junction between the main nail and the cephalomedullary nail. This demonstrated that the area where the cephalomedullary nail passed through the main nail was a stress concentration area. When axial loads of 2100 N were applied, the maximal stress on implants for PFNA, InterTan and NIS was 1257.3 MPa, 1069.2 MPa, and 928.99 MPa. Compared to PFNA, the maximal stress reduction at implants for NIS was 26.1% under axial loads. Besides, the maximal stress at implants was 59.333 MPa, 96.302 MPa, and 104.64 MPa for PFNA, InterTan and NIS under bending loads while under torsion loads it was 512.3 MPa, 477.11 MPa, and 438.16 MPa, respectively. The above data demonstrated that the NIS possessed more uniform stress distribution than PFNA and InterTan for managing UFIFs.

### Stress at bones

**FIGURE 6:**
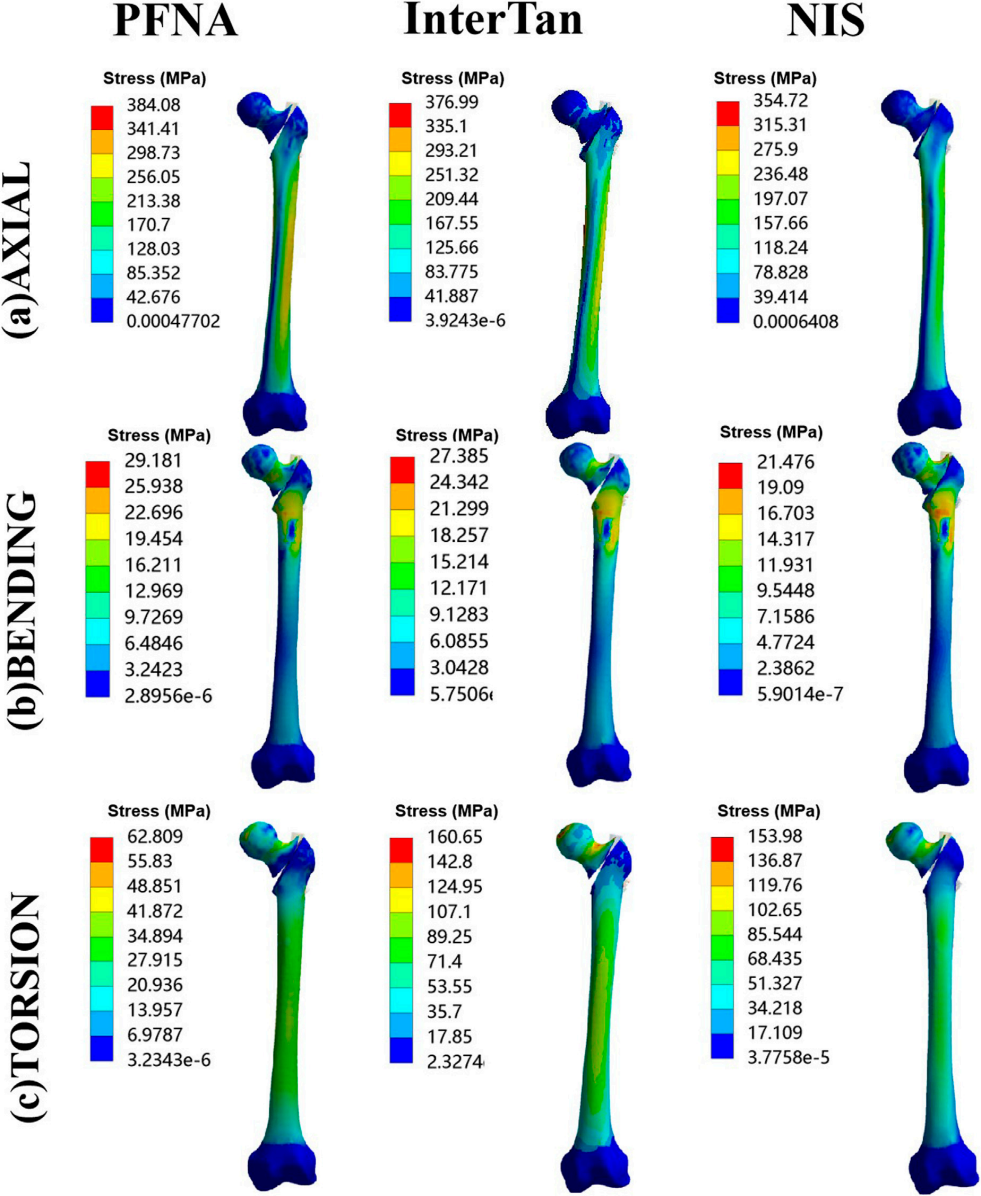
Von Mises stress on bones under axial, bending, and torsion loads for three configurations. Three configurations included PFNA, InterTan, and NIS. PFNA stands for proximal femoral nail antirotation. NIS stands for the new intramedullary system.

Stress at bones for three different models was displayed in Figure 6. When axial loads of 2100 N were applied, from the highest to the lowest stress at bone, the three configurations were sorted as follows: PFNA, InterTan, and NIS. Under bending load conditions, it was also sorted as follows: PFNA, InterTan and NIS. Under torsion load conditions, it was sorted as follows: InterTan, NIS, and PFNA. The maximal stress reduction of the NIS relative to PFNA reached 7.6% under axial loads of 2100 N, and 26.4% under bending loads, respectively. The trend direction of stress on bones was similar to that of stress on implants for UFIFs.

### Comparison of biomechanical properties and statistical analysis

Table 2 displayed statistical analysis data between the PFNA and NIS groups. Five repeated tests were conducted under axial loads of 2100N. The maximal displacement was 13.46 ± 0.57 mm for the NIS group and 17.06 ± 0.67 mm for the PFNA group, with significant difference between the two groups (*p* < 0.05). The values of MDFS were 11.42 ± 0.43 mm and 14.88 ± 0.51 mm for the NIS and and PFNA groups, and the difference was significant (*p* < 0.05). The maximal stress at implants was 927.2 ± 25.7 MPa and 1250.1 ± 47.7 MPa, and there was significant difference between the two groups (*p* < 0.05). The maximal stress at bones was 357.1 ± 16.7 MPa and 381.7 ± 13.3 MPa for the NIS and PFNA groups, and the difference was statistically significant (*p* < 0.05). Therefore, under axial loads of 2100 N, the NIS showed better biomechanical properties than PFNA for managing UFIFs.

**TABLE 2:** Statistical analysis of comparison of biomechanical properties between PFNA and NIS under axial loads of 2100 N.

Biomechanical properties	PFNA	NIS	t	*p*
Maximal displacement (mm)	17.06 ± 0.67	13.46 ± 0.57	9.151	0.000
Maximal displacement of fracture surface (mm)	14.88 ± 0.51	11.42 ± 0.43	11.598	0.000
Maximal stress at implants (MPa)	1250.1 ± 47.7	927.2 ± 25.7	13.322	0.000
Maximal stress at bones (MPa)	381.7 ± 13.3	357.1 ± 16.7	2.577	0.037

Notes: PFNA, stands for proximal femoral nail antirotation; NIS, stands for the new intramedullary system.

## Discussion

Intramedullary fixation methods have been the mainstream management for unstable femoral intertrochanteric fractures because of minimal invasion, mild soft tissue injuries, and central fixation (Haidukewych, 2009; Parker and Handoll, 2010). Among the limited intramedullary implants, PFNA is regarded as the golden standard by lots of scholars for such fractures (Lewis et al., 2022). However, PFNA fixation is accompanied by a high rate of surgical complications, including withdrawal, cut-out, varus collapse (Chapman et al., 2018). The occurrence of these complications puts patients at a relatively high risk of re-operation and mortality. Therefore, designing implants with better biomechanical properties for managing UFIFs has become a research hotspot.

Wang et al. compared the biomechanical properties of PFNA, InterTan, and proximal femur bionic nail (PFBN) for the management of elderly intertrochanteric fractures via finite element method (Wang et al., 2022). Their results indicated that compared to PFNA and InterTan nails, PFBN possesses better biomechanical characteristics. There are two nails, including one pressure nail, and one tension nail in the femoral neck, which are locked at a certain angle, enhancing the biomechanical stability of PFBN. Yet, the medial support effect of PFBN for comminuted intertrochanteric fractures of the medial wall is not yet clear. Regaining medial support is vital for comminuted and unstable intertrochanteric fractures. Yet, it is sometimes unachievable in a specific operation. These comminuted intertrochanteric fractures occupy over eighty percent of unstable intertrochanteric fractures (Grønhaug et al., 2022). As a result, they have been the major cause for implant failures. Therefore, strengthening the support of the medial wall via implants may be a good scheme for such fractures. Nie et al. developed a new intramedullary implant through enhancing medial cortical support to decrease complications for UFIFs via finite element method (Nie et al., 2022). It is called medial sustain nail (MSN). Their research indicated that MSN possesses better mechanical properties than PFNA. The proximal part of this implant has two nails, including one long nail and one short nail. The short nail is located on the inner side, and could provide good medial support. Yet, this design has limited antirotation effect and is prone to cut-out and loosening. Other scholars have used the cerclage cable technique to enhance the medial support of the cephalomedullary nail in patients with intertrochanteric fractures (Kulkarni et al., 2017), but whether this technique could improve fixation is still controversial (Kang et al., 2021; Rehme et al., 2021). Ceynowa et al.‘s study showed that the cerclage cable did not significantly enhance medial support of intertrochanteric fractures via mechanical tests (Ceynowa et al., 2021). Based on these factors, the authors have attempted to develop a new implant to better fix unstable intertrochanteric fractures.

According to traditional biomechanical theory, finite element method could transform the object being studied into a configuration formed by numbers of finite unit combinations, and the mathematical simulation analysis is gotten. The data of the object’s displacement, stress distribution, etc. could be visually reflected the whole or partial mechanical features of the configuration, and different parameters could be timely rectified. Compared to biomechanical experiments, finite element method possesses several advantages, such as short cycles, low costs, and high efficiency (Huang et al., 2015). The authors performed finite element analysis of PFNA, InterTan, and NIS for fixation of AO/OTA 31-A2.3 unstable femoral intertrochanteric fractures. Based on our data, the NIS possessed the best mechanical characteristics, followed by InterTan, and then PFNA under axial, bending, and torsion load conditions. Moreover, after repeating for five times under axial loads of 2100 N, the statistical difference between the PFNA group and the NIS group was significant (*p* < 0.05). The unique design of the NIS might lead to these results. There are three nails in the proximal section of the implant. Two of them are distributed with the included angle of 7.5° in the femoral head. The design of 7.5° ensures that it could provide good antirotation effect while it will not penetrate the femoral neck due to excessive angle. Actually, as shown in the results under torsion loads, it did play a better antirotation effect than the single cephalomedullary nail of PFNA and the parallel and tight design of InterTan. In the PFNA and InterTan models, the junction between the cephalomedullary nail and the main nail is a stress concentration area. In the NIS model, due to the introduction of the subtrochanteric nail, the stress of the implant is dispersed to a certain extent. This reduces the risk of implant failure. In addition, the subtrochanteric nail of NIS is supported under the lesser trochanter, which can play a supporting role for the medial wall of the trochanter. This nail could effectively disperse bending and axial loads. This might enable the NIS to effectively reduce the occurrence of varus deformity in patients with UFIFs.

There are several limitations of this study. The finite element model was applied to compare the mechanical performance of PFNA, InterTan, and NIS for treating UFIFs. The experiment model and analysis process were simplified to a certain extent, and the function of soft tissues and ligaments at bones and implants has not been assessed. Currently, the mechanical evaluation and mathematical simulation of the limb just apply several single direction loads, such as axial, bending or torsion loads. In real life, under physiological conditions, the load acting on the limbs, including the hip joint, is often not a single direction load, but a combination of loads from different directions. The effect of loads on bones and implants may vary depending on the posture and motion states of the limbs. These factors might lead to certain deviations in the experimental results. In addition, the existing conclusions still require further validation through clinical research.

## Conclusion

Compared with PFNA and InterTan nails, the new intramedullary system displayed better mechanical properties under axial, bending, and torsion load conditions. Thus, it may offer a better option for orthopedics to manage unstable intertrochanteric fractures.

## Data Availability

The original contributions presented in the study are included in the article/Supplementary material, further inquiries can be directed to the corresponding authors.
